# What Inspires Biomimicry in Construction? Patterns, Trends, and Applications

**DOI:** 10.3390/biomimetics10050259

**Published:** 2025-04-23

**Authors:** Andrea Goyes-Balladares, Roberto Moya-Jiménez, Víctor Molina-Dueñas, Wilmer Chaca-Espinoza, Teresa Magal-Royo

**Affiliations:** 1 Department of Architecture Graphic Expression, Universitat Politécnica de Valencia, 46022 Valencia, Spain; 2 Faculty of Design and Architecture, Research and Development Directorate, Technical University of Ambato, Ambato 180207, Ecuador; 3 Faculty of Design and Architecture, Technical University of Ambato, Ambato 180207, Ecuador; vh.molina@uta.edu.ec (V.M.-D.); wg.chaca@uta.edu.ec (W.C.-E.); 4 Graphic Engineering Department, Universitat Politécnica de Valencia, 46022 Valencia, Spain; tmagal@upv.edu.es

**Keywords:** biomimicry, biomimetic design, construction technologies, sustainable construction

## Abstract

Biomimicry is redefining the design, architecture, and construction industries by transforming biological principles into innovative solutions that optimize structural, energy, and environmental performance. This study identifies the organisms and natural systems that inspire the industry, establishing patterns, trends, and key applications. Through a systematic literature review, 70 studies documenting bio-inspired applications in materials, structures, and construction systems were analyzed. The findings are organized into a categorization of organisms based on their biological group and a detailed classification according to imitation criteria—form, function, structure, and process—highlighting their applications in the built environment. The results demonstrate the convergence between technology and nature, underscoring the potential of biomimicry for the development of a more sustainable and resilient industry. Furthermore, this study identifies the most recurring sources of inspiration and the main lines of interest in the implementation of biomimetic strategies in construction, consolidating its role as a key tool for the architecture of the future. Based on these findings, the research proposes a biomimetic design framework that aligns architectural needs with suitable imitation strategies and biological analogs, offering a practical tool to guide decision making in the early design phases.

## 1. Introduction

Since the dawn of civilizations, designers, engineers, and architects have significantly contributed to the evolution of the construction industry, developing resilient, functional, and aesthetically pleasing buildings. However, these fundamental requirements must transcend into approaches that include clean technologies, carbon-neutral materials, energy-efficient construction strategies, and lifestyles that respond harmoniously to nature, optimizing the use of renewable resources and strengthening environmental resilience. In recent years, sustainable construction has acquired growing importance, driven by the global urgency to mitigate the negative environmental impacts derived from both the construction process and the production of materials.

In this context, biomimicry has emerged as a revolutionary concept in the construction industry, contributing to the development of solutions based on the adaptation and extraction of ideas from nature, incorporating them into design to help reduce environmental problems. The term biomimicry was conceptualized by the biologist Janine Benyus in 1997, and it refers to a new field of science that studies nature and its models, systems, processes, and elements, imitating them or taking them as a source of inspiration to design sustainable proposals [1]. Biomimicry encourages the transfer of functions, concepts, and strategies from natural organisms and systems to create a more resilient built environment and improve its capacity to develop regenerative systems [2], and it seeks to minimize the negative environmental impact of buildings through greater efficiency and moderation in the use of materials, energy, development space, and the ecosystem in general through a conscious focus on energy conservation and ecology within the design of the built environment [3].

From urban planning and architectural design to construction technologies, materials, and components, nature offers an invaluable model for industry to move toward developing a resilient and environmentally responsible economy. The study of the adaptive mechanisms of natural organisms and systems, refined over millions of years, allows architects, engineers, and technology designers to integrate biomimetic strategies into contemporary construction, optimizing the thermal, structural, and energy performance [4]. Mimicking nature has several levels, including the organismal level, which influences the aesthetic appearance of buildings; the behavioral level, which contributes to energy efficiency and sustainability; and the ecosystem level, which focuses on functional issues at the urban scale [5]. Historically, various civilizations have implemented biomimetic principles in their constructions. The ancient Egyptians, Greeks, and Romans copied natural plants as part of the beauty of their buildings. The Goths combined two levels of biomimicry: the organismal level and the behavioral level. They constructed elegant buildings using elements that mimicked plants, such as the rose window, and other elements inspired by animals, such as flying buttresses and ribbed vaults [6].

In the field of sustainable buildings, the study “Biomimicry in Malaysian Architecture: Crafting A Modular Framework for Sustainable Design” highlights the application of self-healing materials inspired by bone biology and passive ventilation systems based on the morphology of termite mounds, significantly reducing the energy consumption and carbon footprint of buildings [7,8]. Similarly, Ref. [2] analyzes the integration of biomimetic strategies in structural design and material selection, demonstrating how honeycomb-inspired structures and natural ventilation systems can optimize the thermal and energy efficiencies of buildings. In turn, proposals for environmentally efficient solutions are presented to improve thermal comfort and reduce energy consumption through the design of nature-inspired envelopes [9]. Through the analysis of several case studies, the authors identify the application of surfaces inspired by plant stomata to regulate transpiration and dynamic shading systems that mimic the opening and closing of flower petals. They also analyze the use of coral reefs as inspiration for improving structural design, applying biomimetic principles to structural engineering and the development of new construction materials [10]. In a systematic study on biomimicry in sustainable construction, the author mentions that the most common applications include the development of intelligent envelopes inspired by organisms, the use of biomimetic materials that optimize energy efficiency, and the feeding of passive ventilation systems based on natural patterns [11].

The study of biomimicry in construction has found an inexhaustible source of inspiration in natural structures, with a particular emphasis on spider webs and bird and insect nests. The geometry and structural arrangement of spider webs, characterized by a network of radial and spiral filaments, have been the subject of research to improve the fiber orientation in composite materials, optimizing their strength and load distribution in structural applications [12]. In the architectural field, studies have highlighted the importance of natural morphologies in structural resilience, taking as a model the nests of wasps and bees, whose arrangement of hexagonal cells and adherent supports provide structural stability and efficiency [13]. Likewise, studies on the nests of swallows and ovenbirds have shown the influence of available materials in the formation of structures optimized for thermal insulation and mechanical stability [14]. These investigations highlight the potential of integrating biomimetic principles into construction to improve the efficiency and sustainability of one of the most polluting industries.

In recent decades, several architectural projects have applied biomimetic principles to solve structural, thermal, and environmental challenges. A notable example is the Eastgate Centre in Harare, Zimbabwe, which uses passive ventilation strategies inspired by termite mounds, replicating their thermoregulatory processes. Warm air is drawn in through distinctive brick chimneys on the roof, which absorb cool night air from below. Cool air is captured in underground voids and is released into the building during the day through networks of pipes and tunnels. This technique eliminates the need for air conditioning and consumes much less energy [15]. Similarly, the Eden Project in the UK features a geodesic dome structure inspired by the shape and behavior of soap bubbles, replicating their lightweight, modular structure to create biomes composed of hexagonal and pentagonal panels that cluster together as bubbles naturally do. This inspiration allows for construction that is adaptable to the topography of the terrain, with an envelope of inflatable cushions that mimics the insulating and lightweight properties observed in nature in soap bubbles and pollen grains, achieving both structural efficiency and material lightness [16].

The One Ocean Pavilion, built for the Yeosu Expo in South Korea, applies biomimicry by drawing inspiration from the elastic movements of plants, specifically their ability to open and close without hinges, just as flowers and leaves do. This inspiration was translated into a kinetic facade composed of glass fiber-reinforced polymer slats that flexibly deform using actuators, mimicking the natural mechanisms of controlled deformation. The system allows for the creation of animated patterns on the facade, adapts to weather conditions, and offers innovative shading solutions, and it has become a prime example of how the structural and functional principles of nature can be integrated into contemporary architecture [17]. The BIQ House in Hamburg, one of the first bioactive buildings, integrates closed photobioreactors into its facade, a system that mimics natural processes such as photosynthesis and ecological recycling, inspired by the functioning of aquatic ecosystems where microalgae capture CO_2_, generate oxygen, and transform solar energy into biomass [18]. Finally, the Gherkin in London applies biomimicry by drawing inspiration from natural processes to optimize its environmental and structural performance. Its aerodynamic shape reduces wind resistance and improves airflow around the building, mimicking nature’s efficient solutions to external forces. It also incorporates a natural ventilation system using helical atriums and a double-skin facade that allows air circulation and regulates the interior temperature, similar to the functioning of the lungs [19].

Within this framework, this article aims to identify sources of inspiration in the design, architecture, and construction industries by analyzing the biological organisms that are used as references and the imitation criteria that are applied. A systematic approach is established that classifies principles extracted from nature and their application in structures, materials, and construction systems. Through this analysis, we seek to demonstrate how biomimicry contributes to optimizing the energy efficiency, structural strength, and sustainability of buildings, promoting innovative solutions in harmony with the natural environment.

## 2. Materials and Methods

### 2.1. Literature Sources

This study adopted a qualitative and descriptive approach based on a systematic literature review of biomimetic applications in the fields of architecture and construction. Scientific articles were retrieved from five high-impact, peer-reviewed databases: Scopus (n = 28), Web of Science (n = 25), ScienceDirect (n = 10), SpringerLink (n = 4), and IOPscience (n = 3). No records were identified from registers, websites, or citation tracking. Complementary exploratory searches were conducted using Google Scholar and CrossRef, but no additional studies were included from these sources.

The search strategy used keywords such as biomimicry, bio-inspired architecture, biomimetic design, and sustainable construction. The following inclusion criteria were applied:

- Publications from the last ten years (2013–2023);
- Peer-reviewed journal articles, reviews, and documented case studies;
- The explicit application of biomimicry principles in materials, structures, or building systems.

A total of 120 records were initially identified. After removing 15 duplicates, 105 studies were screened by title and abstract, leading to the exclusion of 15 irrelevant articles. The remaining 90 full-text articles were assessed for eligibility. Of these, 20 were excluded for the following reasons: irrelevant content (n = 8), no access to full text (n = 4), duplicate data (n = 3), and methodological issues (n = 5). A final set of 70 studies was included in the qualitative synthesis and analysis.

**Figure 1:**
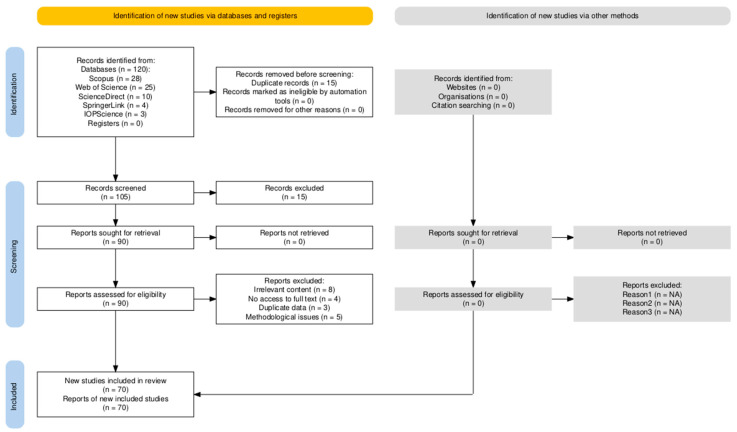
PRISMA diagram for literature inclusion. Diagram generated using the PRISMA 2020 Shiny App tool [20].

To illustrate the study selection process, a PRISMA 2020-compliant flow diagram is provided (Figure 1).

### 2.2. Research Methods and Processes

The analysis of the 70 selected articles was carried out in three structured phases to identify patterns, trends, and relevant applications of biomimicry in architecture:

Phase 1: Compilation and Screening

The bibliographic sources were systematically filtered and evaluated according to the criteria described above. Duplicate entries and off-topic papers were excluded during this phase.

Phase 2: Classification of Imitation Criteria

Each selected article was reviewed to identify the type of organism or system referenced, the element imitated, and the biomimetic strategy applied. The classification was based on four imitation criteria: form (F), function (f), structure (E), and process (P).

Phase 3: Systematization and Synthesis

Applications were organized into eight biological categories: insects, reptiles, plants, marine species, fungi, birds, arthropods, and ecosystems. This classification was developed using a data-driven inductive approach, grounded in a qualitative analysis of the 70 selected studies. As part of this method, biological models were first identified and coded according to their taxonomic and ecological characteristics, and they were then grouped based on the recurring patterns of use and the type of design strategies they have inspired in architectural applications.

Although this typology is original to this study, it aligns with approaches used in previous reviews [11,21], where biological inspirations were also grouped according to their functional impacts on built environments. This classification framework serves as a foundation for identifying patterns and trends in the application of biomimicry across the architectural domain.

To complement this process, a comparative table (Table 1) was developed that relates the studied organisms, the derived biomimetic principles, and their applications in the fields of design and construction. In addition, the frequency of use of the imitation criteria—form (F), function (f), structure (S), and process (P)—in the reviewed studies was analyzed. As part of the analysis, graphical tools such as Sankey diagrams and flowcharts were used to explore the interrelationships between the biological categories, imitation criteria, and areas of application. These resources facilitated data interpretation and provided key inputs for constructing the approach proposed in this study.

## 3. Results

Table 1 summarizes the main organisms and natural systems that have served as a reference in the application of biomimicry in design, architecture, and construction. Eight key categories are presented, detailing the type of organism, the specific part replicated, the imitation criteria applied (form (F), function (f), structure (E), or process (P)), and their respective applications in the industry. This information allows for the analysis of biomimetic-inspired patterns and trends. The table provides a comparative overview that shows how different natural elements have been used to optimize various parameters that guide the industry toward sustainability.

### 3.1. Category Type and Application

In each category, organisms or natural systems have been identified whose characteristics have been replicated in various architectural and construction solutions, following imitation criteria that include form, function, structure, and process.

In the insect category, termite mounds have been widely studied for their passive ventilation systems, inspiring energy-efficient architectural designs [6,21,22,23,24,25,26,27,28,29]. Termite metagenomics has also influenced the development of hydrogen-generating buildings by replicating biological principles across form, function, structure, and process [22]. The dragonfly’s wing geometry has informed the design of lightweight, load-distributing roofs [26,30,31]. Likewise, the fog-harvesting beetle, with its hydrophilic and hydrophobic exoskeleton, has inspired water collection systems in arid regions [25,32,33,34,35,36]. The Saharan silver ant has contributed to radiative cooling technologies through the mimicry of its reflective microscopic hairs [29,37,38,39,40].

Within the reptile category, the structure of snake scales has served as a reference for the design of facades that optimize passive ventilation and solar reflection, with the aim of improving interior thermal comfort [25,41]. In turn, the chameleon has inspired the development of materials capable of dynamic color change through the reorganization of nanocrystals [6,28,29,42], enabling the creation of adaptive windows and facades that regulate their thermal reflectivity based on the outside temperature, thereby reducing energy consumption.

In the plant category, photosynthesis has inspired the design of energy-efficient solar panels and facades [21,41,43,44,45,46,47], while the plant cell wall has served as a model for lightweight yet strong structural materials [21,67]. The lotus leaf’s superhydrophobicity has been replicated in self-cleaning surfaces for windows and cladding [21,24,48,49,50,51,52,53,54,55,56,57]. The flower of *Strelitzia reginae* (Bird of Paradise) has guided the design of adaptive facade shading systems [21,58,59,60,61]. Similarly, the structural flexibility of palm leaves has influenced the development of resilient canopies and modular sunshades [29,62,63,64,65,66]. Pine cones have inspired passive systems for light control, ventilation, and thermal insulation [26].

In the marine species category, the coral reef has served as inspiration for the development of lightweight structures and resistant roofs, due to its porous and branched organization [21,70,71,72,73]. Likewise, the sea urchin skeleton, composed of interconnected polygonal plates, has been replicated in the design of modular structural systems that eliminate the need for torsion elements [74,75,76,77]. The sand dollar, known for its strength, lightness, and efficient use of materials, has served as a reference in the optimization of structures with high mechanical resistance [74]. Furthermore, the translucent gelatinous structure of jellyfish and their ability to interact with light and heat have inspired the development of thermochromic materials, which adjust their light transmission according to the outside temperature [29].

In the fungal category, Pleurotus ostreatus has been analyzed for its biological binding capacity, its adaptation to complex surfaces, and its bioactive mineralization process. These properties have been replicated in the development of innovative biocomposites, such as biodigital bricks, which feature improved structural strength and adaptability [80].

In the bird category, nests have been a source of inspiration for the design of strong and lightweight buildings, based on the intertwined arrangement of branches that optimizes natural ventilation and lighting [13,14,25,81,82]. Furthermore, penguins have been the subject of study due to their thermal regulation capacity, which has allowed for the design of architectural solutions that minimize heat loss in cold climates, improving the energy efficiency of buildings [23,83,84].

In the arthropod category, spider webs have been replicated in the design of facades and flexible roofs due to their high resistance and ability to absorb and distribute energy. These principles have been applied to optimize passive ventilation and improve the structural performance in buildings [12,23,26].

Finally, in the ecosystem category, forests, wetlands, and rivers have been analyzed as models of systemic organization and functioning. The ability of these environments to capture and convert solar energy, store water, and participate in natural cycles such as those of carbon and nitrogen has been replicated in sustainable solutions for urban design. These strategies have enabled the implementation of green infrastructure systems that promote environmental regeneration and resource efficiency in urban environments [23,24,25,26,29,85].

#### Connections Between Architectural Applications, Biological Categories, and Imitation Criteria

**Figure 2:**
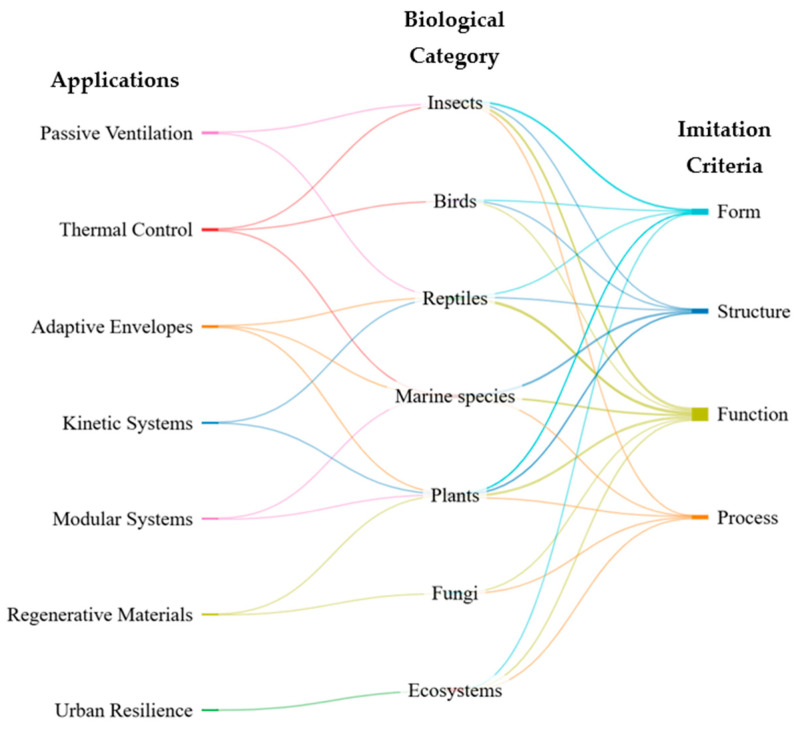
Interconnected flow between applications, imitation criteria, and biological categories in biomimetic design.

Figure 2 presents a tripartite relational structure that allows us to visualize the flow of biomimetic inspiration, from architectural functional needs to defined imitation criteria, passing through specific biological categories. Organized from left to right, the diagram connects seven key applications, with the organisms grouped into seven biological groups, which, in turn, lead to the selection of biomimetic criteria.

Unlike a hierarchical representation, this visualization operates as a system of multiple correspondences, where each trajectory indicates a possible transfer path from biology to architectural design. For example, passive ventilation links to insects and reptiles, leading to the criteria function, form, structure, and process, demonstrating a focus on passive ventilation strategies, natural thermal regulation, and efficient structural organization. Thermal control, moreover, links to insects, birds, and marine species, relating primarily to function, form, and process. This reflects a convergence of natural solutions for dissipating heat, thermal insulation, or responding to environmental conditions. In the case of adaptive envelopes, inspiration comes from plants, reptiles, and marine species and focuses on the criteria function, form, and structure, highlighting the development of enclosures that actively adapt to environmental changes through intelligent morphologies and reactive materials.

Kinetic Systems are linked to plants and reptiles, focusing on the criteria function, form, and structure, suggesting the implementation of dynamic mechanisms inspired by natural movements, such as the opening of flowers or changes in skin color. Modular Systems connect with plants and marine species and relate to function and structure, reflecting the principles of structural repetition, assembly, and spatial adaptability. As for Regenerative Materials, their relationship with fungi and plants activates the criteria function and process, which refer to the development of biocomposites, bioreactors, and photosynthetic materials with the capacity for regeneration, adaptation, and self-repair. Finally, Urban Resilience is linked exclusively to ecosystems, connecting with the criteria function, form, and process, which implies a systemic view of urban design, focused on the integration of natural processes such as water cycles, carbon sequestration, and thermal regulation.

From a critical perspective, the diagram reveals an uneven distribution in the use of imitation criteria. A clear predominance of function and structure is observed, while process, despite its enormous potential in terms of regenerative design and environmental resilience, continues to have a more limited presence in the applications analyzed. This imbalance suggests that strategies based on natural processes, such as self-regulation, photosynthesis, and biological repair, have not yet been fully integrated into architectural practice. Likewise, a strong reliance on widely documented biological groups, such as insects, plants, and reptiles, is identified, which may reflect their extensive empirical base, ease of abstraction, or conceptual familiarity within design. However, other groups such as fungi and ecosystems, although present, are represented in only one application each, suggesting considerable scope for exploration. In contrast, marine species, although less referenced in classical studies, appear to be associated with three distinct applications, demonstrating their growing relevance in modular, adaptive, and thermoregulatory solutions.

Overall, this visualization offers a valuable tool not only for understanding the current connections between biology and the construction industry but also for identifying conceptual gaps and opportunities for expansion toward a more diverse, ecosystem-based, and regenerative design-oriented approach.

### 3.2. Imitation Criteria by Category

**Figure 3:**
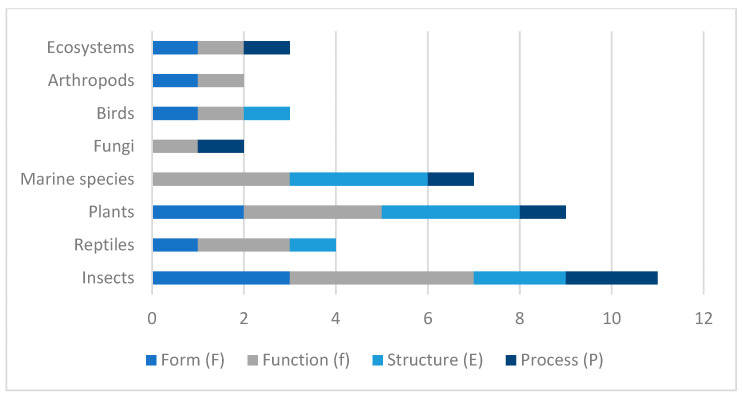
Distribution of imitation criteria by biological category.

The imitation criteria established in the different categories and types of organisms or natural systems show a differential distribution in the imitation of characteristics applied in the fields of design, architecture, and construction. Comparing these criteria across the different categories reveals patterns that highlight a preference for some in bio-inspired design (see Figure 3).

In the insect category, the imitation criterion related to function (f = 4) is the most frequently present, followed by form (F = 3), structure (E = 2), and process (P = 2), as evidenced by applications such as termite mounds, dragonfly wings, and beetle exoskeletons [6,22,36]. In the reptile category, the most representative imitation criteria are function (f = 2) and form (F = 1), while structure (E = 1) and process (P = 0) are less relevant, based on biomimetic strategies derived from snake scales and chameleon skin [25,41,42]. For plants, a balanced distribution of the criteria is shown, with a high presence in function (f = 3), structure (E = 3), and form (F = 2), while process is less represented (P = 1), reflecting inspiration from lotus leaves, photosynthesis, cell walls, and pinecones [24,41,43,44,45,46,47,48,49,50,51,52,53,54,55,56,57,67,68,69]. Marine species show a significant preference in imitating function (f = 3) and structure (E = 3), with a lower presence in process (P = 1) and an absence of imitation in form (F = 0), as illustrated by applications based on coral reefs, echinoderms, and jellyfish [21,29,70,77]. Fungi present an application through the criteria function (f = 1) and process (P = 1), while form and structure (F = 0, E = 0) do not show significant records, particularly from the species Pleurotus ostreatus [80]. Birds present an equal distribution in the criteria form (F = 1), function (f = 1), and structure (E = 1), while the process is not represented (P = 0), as shown in studies on nest morphology and penguin thermal regulation [13,14,23,25,81,82,83,84]. In the arthropod category, largely represented by spider webs, low values are identified in all the criteria, with a slight predominance in form and function (F = 1, f = 1), while structure and process do not present values (E = 0, P = 0) [12,23,26]. Finally, ecosystems show a homogeneous distribution in all the criteria, with low values in form, function, and process (F = 1, f = 1, P = 1) and an absence of imitation in structure (E = 0), based on systemic inspirations from forests, wetlands, and rivers [23,24,25,26,29,85].

In comparative terms, the results indicate that function is the most frequently replicated biomimetic criterion across all the biological categories (f = 2.5), followed by structure (E = 1.5), form (F = 1.25), and, to a lesser extent, process (P = 0.75). This trend, already evident in the previous graphical analysis, is verified again in this section, reaffirming that biomimicry in design, architecture, and construction is predominantly applied to improve functional performance, particularly with regard to energy efficiency, thermal regulation, structural resilience, and adaptive capacity in the face of changing climatic conditions.

This emphasis on functional mimicry aligns with global efforts to optimize the performance of the built environment and reduce resource consumption. However, the persistent underrepresentation of the process criterion once again highlights a significant gap in the exploration of biomimetic strategies based on complex biological dynamics, such as self-healing, regeneration, and adaptive transformation. Therefore, expanding the application of the structure and, especially, process criteria could substantially enrich the repertoire of biomimetic solutions available to architects and engineers, promoting more resilient, circular, and regenerative approaches.

### 3.3. Patterns and Trends Identified

**Figure 4:**
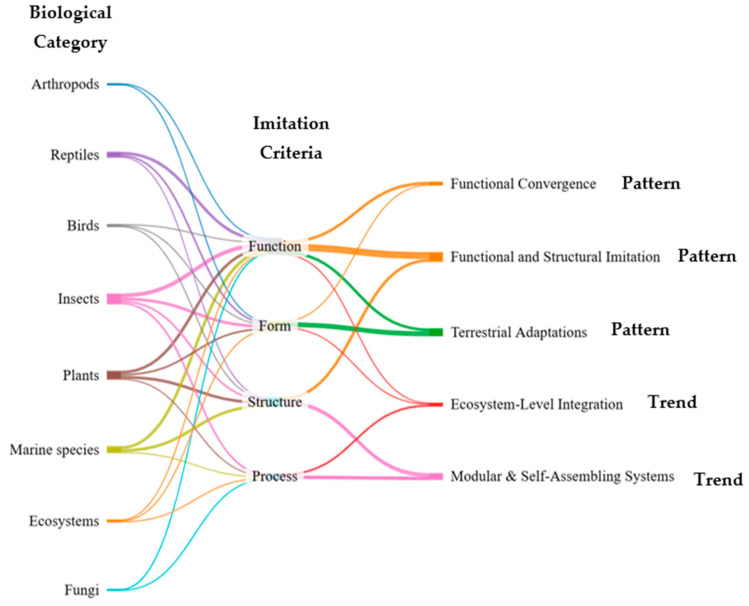
Interconnected flow of biological categories, imitation criteria, and patterns and trends.

Figure 4 shows the interconnected relationships between the biological categories, imitation criteria, and main patterns and trends identified in the construction industry. The diagram represents how eight distinct biological sources contribute to the four main imitation criteria, which, in turn, relate to different application types, organized into three observed patterns—functional and structural imitation, functional convergence, and terrestrial adaptations—and two emerging trends—modular and self-assembling systems, and ecosystem-level integration [6,12,13,14,21,23,24,25,26,29,57,70,71,72,74,75,76,77,78,79,80]. The width of each connection reflects the relative frequency and strength of the association observed across the reviewed studies. Notably, the predominance of function and structure as imitation criteria supports a performance-driven design paradigm, while the consistent recurrence of specific biological categories—particularly insects and plants—highlights their relevance in current biomimetic strategies [21,22,23,24,25,26,27,28,29,30,31,41,42,43,44,45,47,48,49,50,51,52,53,54,55,56,57].

This systematic review thus revealed both recurrent patterns in how biomimicry is applied in architectural design and emerging trends that suggest a directional evolution in the field. The Sankey diagram (Figure 4) effectively maps the flow of relationships between biological models, design logics, and strategic outcomes, offering a framework for understanding how nature-inspired principles are currently being integrated into architecture.

The analysis of biomimetic applications in architecture and construction reveals a set of recurring patterns and emerging trends that reflect how natural principles are interpreted and adapted in design. The following sections synthesize the main findings into two perspectives: first, the observed patterns, which highlight the frequent strategies and biomimetic logics already present in the reviewed studies; and second, the emerging design trends, which point to the evolution of biomimicry toward modularity, regeneration, and ecosystem-level integration. This discussion aims to connect the biological sources of inspiration with their practical implications, emphasizing both current practices and future directions in the field.

#### 3.3.1. Observed Patterns

Dominance of Functional and Structural Imitation

A prominent pattern is the repeated presence of function and structure as the dominant imitation criteria across most of the biological categories analyzed. These criteria stand out over others, such as form and process, reflecting a focus on optimizing architectural performance, particularly in aspects such as thermal regulation, structural strength, and energy efficiency. Notable examples of this trend include passive ventilation strategies inspired by termite mounds and the structural logic replicated from sea urchin shells [22,70].

Functional Convergence Across Biological Categories

Another recurring pattern is the convergence of functions inspired by unrelated species. Organisms from different taxonomic groups—such as pine cones, echinoderms, fog-harvesting beetles, and coral reefs—have inspired similar architectural solutions, like climate-responsive facades and atmospheric water collection systems [21,29,37,73]. This cross-domain convergence underscores the transdisciplinary potential of biomimetic design.

Emphasis on Terrestrial Adaptations

A third key pattern is the predominant inspiration from terrestrial organisms, notably insects, reptiles, and birds. These species offer biologically evolved solutions for high-thermal-variability environments, such as snake-scale heat dissipation and chameleon skin reflectivity, which inform the design of thermally adaptive envelopes and passive cooling systems [25,41,42,81,82]. This reflects a preference for organisms from fluctuating terrestrial climates over those from stable marine ecosystems.

#### 3.3.2. Emerging Design Trends

Modular and Self-Assembling Systems

An emerging trend is the increasing interest in modular and self-assembling systems, inspired by biological models such as fungi, corals, and echinoderms. These organisms demonstrate structural repetition, regeneration, and material efficiency, informing the creation of lightweight, adaptive, and sustainable architectural components [74,77,80]. The use of modular logic enables scalability, repairability, and resource efficiency, aligning with circular design principles.

In addition to their structural benefits, these systems support essential circular economy practices, such as disassembly, reuse, and material regeneration. Advances in additive manufacturing have enabled the production of prefabricated components—such as 3D-printed panels modeled on echinoderm skeletons or interlocking bricks made from mycelium-based composites—that simplify construction logistics and significantly reduce waste. These biomimetic modules are especially valuable for scalable infrastructure, emergency shelters, and adaptable urban housing, where rapid deployment and environmental responsiveness are critical.

Ecosystem-Level Integration

At a broader scale, biomimicry is evolving toward ecosystem-based thinking. Inspired by forests, wetlands, and mycelial networks, architects are designing systems that replicate the regenerative cycles and organizational logic of natural ecosystems [23,24,25,26,29,85]. These strategies foster Urban Resilience through green infrastructure capable of managing water filtration, thermal control, and pollutant absorption, moving from isolated biomimetic solutions toward holistic environmental integration.

Ecosystem-level integration also presents new opportunities for embedding dynamic environmental intelligence into the built environment. By emulating the interconnected processes found in nature—such as nutrient cycling, carbon sequestration, and water regulation—architectural projects can function as self-regulating systems. Notable examples include bio-integrated urban wetlands for flood mitigation, living walls that purify indoor and outdoor air, and facade systems that emulate forest canopy layers to control light, airflow, and temperature. These approaches not only enhance environmental performance but also promote biodiversity and improve human well-being in high-density urban contexts.

## 4. Discussion

The results of this study confirm that biomimicry has been widely explored and applied in the fields of design, architecture, and construction, with a predominant focus on the functional and structural optimization of materials and construction systems. The identification and analysis of natural organisms and systems have allowed for the construction of a comparative framework where imitation criteria (form, function, structure, and process) serve as a basis for evaluating the transferability of adaptive strategies to the built environment. A clear inclination is evident toward the reproduction of functions that improve energy efficiency, ventilation control, and mechanical resistance, frequently surpassing those proposals of an aesthetic nature or focused on biological processes. This is directly related to the argument presented in the Introduction, which argues that biomimicry represents a way to optimize the thermal, structural, and energy performance in contemporary architecture.

However, barriers remain that limit the widespread implementation of biomimicry in architectural practice. These include the lack of standardized design methodologies, the high costs of bio-inspired materials, and the absence of specific regulatory frameworks. Overcoming these obstacles will involve fostering interdisciplinary collaboration, generating institutional incentives, and structurally incorporating biological knowledge into the training of designers and architects.

In conclusion, biomimicry is reaffirmed not only as a source of inspiration but also as a strategic tool for reconfiguring the built environment. Its true potential lies in connecting biology with technology, facilitating the development of intelligent, efficient, and regenerative architectural systems that respond to the challenges of contemporary environmental sustainability.

### 4.1. Applied Perspectives on Biomimicry

Building on the patterns and trends identified in the previous section, this subsection explores the practical implications of biomimetic strategies in architectural design. It highlights key examples of functional convergence, technological innovation, and opportunities for systemic ecological integration.

One of the most significant aspects is the functional convergence between organisms from different biological categories. Examples such as the fog-harvesting beetle and coral reefs have served as inspiration for the design of water-harvesting systems in arid and urban contexts. This convergence demonstrates that biomimetic innovation does not depend exclusively on the type of organism but rather on transversal biological principles that can be adapted to multiple challenges of the built environment. Such an approach confirms the methodological flexibility of biomimicry, which focuses on performance and adaptability. This logic is manifested in cases such as the Eden Project, whose modular geodesic structure is inspired by the morphology of soap bubbles, or the BIQ House, which reproduces the biochemical processes of microalgae to generate energy and regulate the climate using photobioreactors.

The literature reviewed also shows how biomimicry has driven disruptive innovations, such as lotus leaf-inspired superhydrophobic surfaces for self-cleaning applications, or Pleurotus fungus-based biocomposites used in the production of biodegradable bricks with greater thermal insulation. Likewise, plant cellular structures and echinoderm skeletons have inspired modular components and self-assembling systems for prefabricated construction, promoting adaptive and sustainable solutions. The case of the One Ocean Pavilion reinforces this perspective, integrating natural opening and closing mechanisms, such as those of flowers and leaves, into a kinetic and adaptable facade, a tangible example of how functional and structural principles can be combined with natural dynamic processes.

Despite these advances, there is limited application of strategies focused on dynamic biological processes, such as self-healing, real-time adaptation, and metabolic regulation. These natural mechanisms could enable the development of smart materials and construction systems capable of responding to environmental conditions without requiring external energy. The lack of exploration of these functions represents a strategic opportunity for future research and technological development.

The evolution from isolated biomimetic applications toward a systemic vision of ecological integration is also notable. Various urban design strategies already reproduce processes observed in ecosystems such as forests, wetlands, and coral reefs, such as water filtration, thermal regulation, and pollutant absorption. This approach promotes regenerative urban planning, in which cities function as self-regulating ecosystems. This approach is consistent with the trends detected in the results (Section 3.3.2), which point to the integration of solutions inspired by the organizational logic of ecosystems as part of environmentally responsible architecture.

### 4.2. Proposed Framework for Implementing Biomimicry in Construction Industry

Building upon the insights gathered from the systematic review presented in previous sections, this study proposes an operational framework designed to support the implementation of biomimicry in architectural design and construction. The framework synthesizes the key variables identified in the literature—such as architectural needs, imitation criteria, biological categories, and representative organisms—into a visual and functional flow that facilitates informed decision making during the early stages of design.

The objective of this framework is to help designers align specific architectural challenges with appropriate biomimetic strategies by tracing a logical path from practical needs (e.g., thermal regulation, structural lightness, moisture control) to the type of imitation required (form, function, structure, or process), and finally, to the most relevant biological categories and organisms that have demonstrated analogous capacities in nature. This structured logic fosters a more consistent and intentional approach to biomimetic innovation in architecture, allowing for both creative exploration and technical justification.

**Figure 5:**
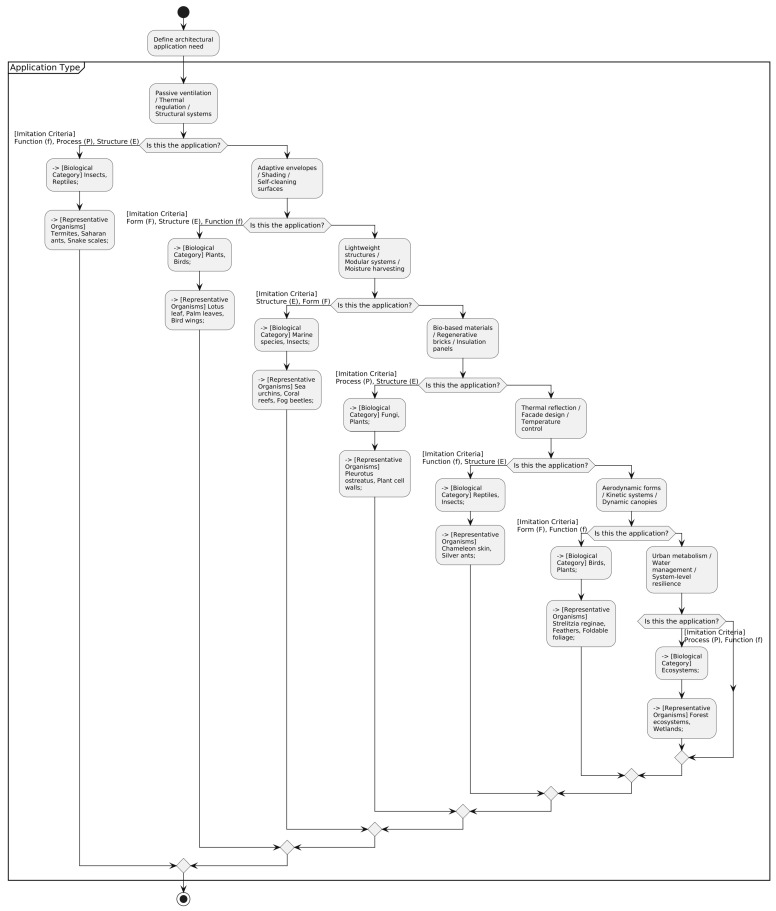
Decision-making flowchart for the selection of biomimetic criteria and biological models based on architectural application needs.

Figure 5 illustrates the proposed flowchart, which maps the relationships between the application types, imitation criteria, biological categories, and representative species. The diagram is designed to be used as a practical tool in the early design phases, where architects and researchers can define a performance-based need and follow a guided path toward identifying natural models that have inspired successful solutions in similar contexts. For instance, if a design team seeks to address passive thermal regulation, the diagram directs them to consider criteria such as function and structure and biological analogues like termite mounds or Saharan ants, which have informed naturally ventilated architectural solutions in extreme climates.

By operationalizing the patterns and trends identified in the literature review, this framework bridges the gap between academic theory and applied design methodology, offering a replicable approach that supports both performance optimization and ecological integration in the built environment.

## 5. Conclusions

The patterns observed in the application of biomimicry in the fields of design, architecture, and construction reflect a predominant focus on the optimization of function and structure, with a marked functional convergence between different organisms and natural systems. The sector’s prioritization is evident in the trend toward bio-inspired strategies that improve the energy efficiency, mechanical resistance, and climatic adaptability of buildings, rather than focusing solely on formal principles. The trend toward the adoption of modular principles, thermal regulation inspired by terrestrial organisms, and the integration of ecosystem models into urban planning suggests an evolutionary path toward more adaptive, efficient, and sustainable architectures.

The distribution of the imitation criteria shows that certain organisms and natural systems have been emulated primarily in function and structure, while the imitation of processes and forms is less common. These findings highlight the relevance of biomimicry as an innovation strategy in architecture and construction, enabling the development of resilient solutions based on biological principles optimized throughout natural evolution. However, there are still opportunities for exploration in the replication of dynamic self-regulation mechanisms and the refinement of adaptive design approaches. The implementation of bio-inspired materials with regenerative properties, the integration of ventilation and air conditioning systems based on natural models, and the exploration of self-assembling patterns in architectural structures emerge as lines of research with high potential for impact on the sustainability of the built environment.

Nonetheless, this study presents certain limitations. The literature review was based on sources indexed in selected databases, which may have excluded relevant but non-indexed or emerging publications. The qualitative classification by imitation criteria and biological category provides a comparative framework but does not quantify the performance outcomes of each strategy. Additionally, this study does not include empirical case analyses or simulation-based validation. Future research would benefit from incorporating quantitative performance data, life-cycle assessments, and the integration of biomimetic applications in real-world architectural projects.

Likewise, this study does not address the economic or technical feasibility of biomimetic strategies. Its focus is primarily on identifying what organisms inspire design, what aspects are imitated (form, function, structure, process), and how these are applied in architectural practice. Future investigations should evaluate cost-effectiveness, scalability, and regulatory barriers to enhance the practical implementation of biomimicry in large-scale construction.

## 6. Future Directions

The future of sustainable construction requires an integrated approach, wherein biomimicry—supported by advances in materials science, computational modeling, and digital manufacturing—guides the development of smarter, more sustainable architectural solutions. Future research should explore the use of living systems in buildings, AI-assisted design, and materials with adaptive or self-healing properties.

Despite its growth, biomimicry still faces important challenges. Process-level mimicry is underdeveloped, and many proposals lack real-world testing or measurable performance data, making comparisons difficult. Ecosystem-based approaches remain mostly theoretical due to high costs, scalability issues, and the absence of clear methodologies.

To move forward, it is vital to develop responsive materials and systems that interact with the environment without external energy, and to validate them through real-world testing. AI and digital tools offer new possibilities to simulate natural processes and translate them into efficient, buildable solutions. Technologies like 4D printing and generative design can help create components that adapt and self-assemble.

At the urban scale, nature-based planning inspired by forests, wetlands, or coral reefs can shape cities with circular systems and decentralized infrastructures. Achieving this will require clear regulations, design standards, and collaboration across disciplines, positioning biomimicry as a key strategy for sustainable architecture.

## Figures and Tables

**Table 1:** Sources of inspiration and criteria for imitation in the design, architecture, and construction industries.

Category	Type of Organism or Natural System	Part of the Organism or Natural System	Reference for Imitation	Imitation Criteria				Application	Sources
				F	f	E	P		
Insects	Termite mounds	-	Thermal regulation and passive ventilation system			x	x	Ventilation, thermal regulation, and energy efficiency in buildings	[6,21,22,23,24,25,26,27,28,29]
	Termites	Appearance and digestive system	Metagenomics	x	x	x	x	Buildings capable of efficiently producing hydrogen using metagenomic principles	[22]
	Dragonfly	Wings	Geometric patterns	x	x			Lightweight and resistant roofs	[26,30,31]
	Fog-harvesting beetle	Exoskeleton	Hydrophilic and hydrophobic surfaces		x			Water collection from fog or humid air through condensation	[25,32,33,34,35,36]
	Sahara silver ant	Microscopic triangular hairs	Reflective, pristine structures	x	x			Radiative cooling systems	[29,37,38,39,40]
Reptiles	Snake	Scales	Passive ventilation and solar reflection	x	x	x		Facades that optimize ventilation and solar reflection	[25,41]
	Chameleon	Ability to change skin color in response to external stimuli	Dynamic adaptation to the environment and nanocrystal reorganization		x			Reduction in energy consumption; increased indoor thermal comfort; adaptive window systems	[6,28,29,42]
Plants	General plants	Photosynthesis	Solar energy capture and utilization				x	Solar panels, energy-efficient facades	[21,41,43,44,45,46,47]
	Lotus flower	Leaf	Superhydrophobicity		x			Self-cleaning windows and coatings	[23,24,48,49,50,51,52,53,54,55,56,57]
	Strelitzia reginae (Bird of Paradise)	Leaves	Flower opening and closing mechanism during pollination		x			Adaptive shading on facades	[21,58,59,60,61]
	Palm trees and tropical plants	Leaves	Structural flexibility	x		x		Flexible and resistant shading systems; dynamic roofs; modular sunshades	[29,62,63,64,65,66]
	Pine	Pinecones	Ability to open and close depending on environmental humidity		x			Systems that automatically adjust to regulate light entry, ventilation, or thermal insulation without the need for electricity	[26]
	Cell wall	-	Structure and resistance			x		Material optimization in lightweight yet resistant structures	[24,67]
	General trees	Structure	Multilayer configurations	x				Adaptive kinetic facades	[29,68,69]
Marine species	Coral reef	Porous and branched structure	Strength and lightness			x		Lightweight structures and resistant roofs	[21,70,71,72,73]
	Sea urchin	Skeleton	Modular polygonal plates connected by calcareous projections			x		Resistant and lightweight structures; joints that withstand shear and normal forces, eliminating the need for torsion elements	[74,75,76,77]
	Sand dollar	Plates	Strength, lightness, and material efficiency			x		Material efficiency in lightweight and resistant structures	[74]
	Algae	-	CO_2_ absorption and oxygen production during growth		x			Bio-facades with bioreactors for thermal regulation and CO_2_ capture	[23,78,79]
	Jellyfish	Translucent gelatinous structure and ability to interact with light and heat	-		x		x	Thermochromic materials that adjust their light transmission based on the external temperature	[29]
Fungi	Pleurotus ostreatus	-	Biological binding capacity, adaptation to complex surfaces, bioactive mineralization processes		x		x	Design of innovative biocomposites, such as biodigital bricks	[80]
Birds	Nest	Interwoven branches	Adaptation to changing climate conditions for optimal lighting and natural ventilation	x		x		Strong, lightweight buildings that optimize climatic conditions	[13,14,25,81,82]
	Penguins	-	Process for adapting to climatic conditions		x			Solutions that minimize heat loss in structures	[23,83,84]
Arthropods	Spiders	Web	Strength and flexibility and ability to absorb and distribute energy	x	x			Optimization of passive ventilation and structural resistance; flexible facades and roofs	[12,23,26]
Ecosystems	Forests, wetlands, rivers, and local biodiversity areas in urban environments	-	Ecosystem organization and ecosystem functioning to capture and convert solar energy, store water, and participate in natural cycles (carbon, nitrogen)	x	x		x	Sustainable, efficient, and regenerative solutions	[23,24,25,26,29,85]
